# Neuroprotection with metformin and thymoquinone against ethanol-induced apoptotic neurodegeneration in prenatal rat cortical neurons

**DOI:** 10.1186/1471-2202-13-11

**Published:** 2012-01-19

**Authors:** Ikram Ullah, Najeeb Ullah, Muhammad Imran Naseer, Hae Young Lee, Myeong OK Kim

**Affiliations:** 1 Department of Biology, College of Natural Sciences (RINS) and Applied Life Science (BK 21), Gyeongsang National University, Jinju, 660-701, Republic of Korea; 2 Institute of Basic Medical Sciences, Khyber Medical University, Peshawar, Khyber Pakhtoonkhwa, Pakistan; 3 Center of Excellence in Genomic Medicine (CEGMR), King Abdulaziz University, Jeddah, Saudi Arabia

## Abstract

**Background:**

Exposure to ethanol during early development triggers severe neuronal death by activating multiple stress pathways and causes neurological disorders, such as fetal alcohol effects or fetal alcohol syndrome. This study investigated the effect of ethanol on intracellular events that predispose developing neurons for apoptosis via calcium-mediated signaling. Although the underlying molecular mechanisms of ethanol neurotoxicity are not completely determined, mitochondrial dysfunction, altered calcium homeostasis and apoptosis-related proteins have been implicated in ethanol neurotoxicity. The present study was designed to evaluate the neuroprotective mechanisms of metformin (Met) and thymoquinone (TQ) during ethanol toxicity in rat prenatal cortical neurons at gestational day (GD) 17.5.

**Results:**

We found that Met and TQ, separately and synergistically, increased cell viability after ethanol (100 mM) exposure for 12 hours and attenuated the elevation of cytosolic free calcium [Ca^2+^]_c_. Furthermore, Met and TQ maintained normal physiological mitochondrial transmembrane potential (Δψ_M_), which is typically lowered by ethanol exposure. Increased cytosolic free [Ca^2+^]_c _and lowered mitochondrial transmembrane potential after ethanol exposure significantly decreased the expression of a key anti-apoptotic protein (Bcl-2), increased expression of Bax, and stimulated the release of cytochrome-c from mitochondria. Met and TQ treatment inhibited the apoptotic cascade by increasing Bcl-2 expression. These compounds also repressed the activation of caspase-9 and caspase-3 and reduced the cleavage of PARP-1. Morphological conformation of cell death was assessed by TUNEL, Fluoro-Jade-B, and PI staining. These staining methods demonstrated more cell death after ethanol treatment, while Met, TQ or Met plus TQ prevented ethanol-induced apoptotic cell death.

**Conclusion:**

These findings suggested that Met and TQ are strong protective agents against ethanol-induced neuronal apoptosis in primary rat cortical neurons. The collective data demonstrated that Met and TQ have the potential to ameliorate ethanol neurotoxicity and revealed a possible protective target mechanism for the damaging effects of ethanol during early brain development.

## Background

Exposure to ethanol during brain development might provoke neurodevelopmental defects that are referred to as fetal alcohol effects (FAE) or fetal alcohol syndrome (FAS) [1]. Ethanol potentially damages the developing brain by affecting neurogenesis, cell migration, or cell survival via different intracellular signaling pathways in prenatal rat cortical and hippocampal neurons [2-4]. Neurodegeneration presents with reduced brain mass and neurobehavioral disturbances in many neurological disorders, and cell death processes are associated with the activation of caspase-3, an executioner protease that is active during apoptotic cell death [5,6]. Apoptosis is a normal process in the developing brain; for optimal development, more than 50% of the original neurons must undergo programmed cell death or apoptosis [7]. Apoptosis is an indispensable mechanism for cellular migration and differentiation during the formation of the developing central nervous system. In particular, Bax and Bcl-2 are major apoptotic proteins and act as an apoptotic inducer and inhibitor, respectively [8,9].

Mitochondria play an important role in apoptosis under a variety of pro-apoptotic conditions, such as oxidative stress and cytochrome-c release. The latter is a key event in the activation of caspase-3, a pivotal downstream step in apoptosis initiation [10,11]. Research has demonstrated protective roles for vitamin E and brain derived-neurotrophic factor (BDNF) against ethanol-induced apoptosis, which causes the generation of reactive oxygen species (ROS) and disrupts mitochondrial membrane potential (Δψ_M_) [12]. Cellular or sustained Ca^2+ ^overload is also an important mechanism that contributes to ethanol-induced programmed cell death through the activation of post-mitochondrial events involving caspase activation [13,14].

Different therapeutic approaches have been investigated to overcome neurodegenerative diseases [15]. Some approaches consist of drugs that act directly on mitochondrial function, such as cyclosporine A, which is a permeability transition pore (PTP) reference blocker [16]. It was reported that the antidiabetic drug metformin (Met) prevents PTP opening and subsequent cell death in various endothelial cell types exposed to high glucose levels [17]. Recently, it was also reported that Met can interrupt the apoptotic cascade in a model of etoposide-induced cell death by inhibiting PTP opening, blocking the release of cytochrome-c and preventing subsequent cell death [18]. This finding is of great clinical interest because Met, beyond its anti-hyperglycemic function, can also act as a pharmacological agent in the treatment of neurodegenerative disorders. In this study, we showed that Met is substantially neuroprotective at a therapeutic dose against ethanol-induced cell death in rat primary cortical neurons.

Thymoqinone (TQ), the active component of *Nigella sativa *(NS) seeds, has broad and versatile pharmacological effects. These effects include strong antioxidant activity against free radical-generating agents, such as doxorubicin-induced cardiotoxicity [19,20]. Recently, it was reported that NS extract and TQ protect against cell death induced by serum/glucose deprivation in PC12 cells via a direct reduction in intracellular ROS [21] and in chronic toluene-induced neurodegeneration in the hippocampus [22]. Recent studies reported that TQ prevented brain damage in a model of transient forebrain ischemia in the rat hippocampus. TQ stimulated resistance to oxidative stress by decreasing the elevated levels of malondialdehyde, glutathione (GSH) contents, catalase and superoxide dismutase (SOD) [23]. A protective role of TQ was reported in 1-methyl-4-phenylpyridinium (MPP)-treated primary dopaminergic cultures and a primary Parkinson's disease model involving rotenone and neuroinflammatory mechanisms [24].

Previously, we reported that antioxidant vitamin C was neuroprotective against ethanol and nicotine via the modulation of GABA_B _receptor and protein kinase A (PKA-α) expression in prenatal and postnatal rat brain [25,26]. Thus, this study was designed to investigate the molecular mechanisms involved in TQ and Met neuroprotection against ethanol-induced apoptosis. We investigated how Met and TQ regulate Ca^2+ ^dysregulation, mitochondrial dysfunction, cytochrome-c release, caspase activation and the Bcl-2 family of proteins during ethanol-induced neuronal apoptosis.

## Results

### Effect of TQ and Met on ethanol-induced cell death in primary rat cortical neurons

**Figure 1:**
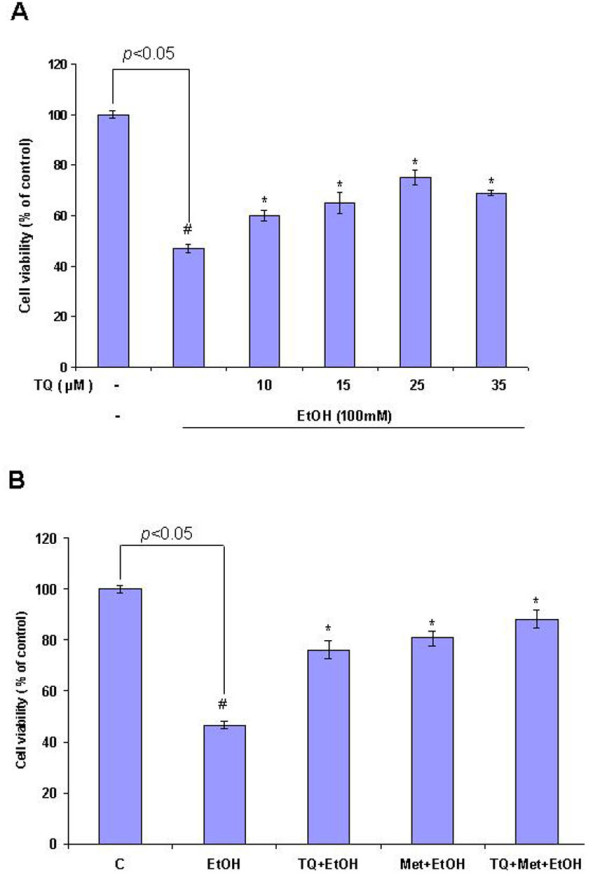
**Met and TQ prevent ethanol-induced neurotoxicity in cultured cortical neurons**. **(A) **MTT assay of cell viability in primary fetal rat cortical neurons treated with 100 mM ethanol (EtOH) for 12 h. Cells were treated for 12 h with normal media as control (C), ethanol (EtOH, 100 mM), TQ +EtOH (TQ: 10, 15, 25 and 35 μM), TQ (25 μM) plus ethanol (TQ+EtOH), Met (10 mM) plus ethanol (Met+EtOH), TQ plus Met plus ethanol (TQ+Met+EtOH), respectively. **(B) **Percentage of cell viability with selected concentrations of TQ (25 μM), 10 mM Met, 100 mM EtOH; in all experiments, TQ and Met were co-incubated with ethanol for a 12 hours time period. Data are the mean ± SEM of three independent experiments (*n = *3), with 3 plates in each experiment. Symbols: ^#^*P *< 0.05 significantly different from control; *P < 0.05 different from ethanol.

Cortical neurons were treated with ethanol (100 mM), TQ (10, 15, 25 and 35 μM) and Met (10 mM) in different combinations for 12 h, and cell viability was measured by 3-[4,5-dimethylthiazol-2]-2,5 diphenyltetrazolium bromide (MTT) assay. TQ and Met were co-incubated with ethanol in all experimental groups for 12 h. Cortical neurons exposed to ethanol exhibited a decrease in cell viability after 12 h incubation, but cotreatment with TQ at different concentrations reversed cell loss. All of the TQ concentrations tested were effective, but 25 μM was the most efficacious (Figure 1A). In the context of ethanol treatment, cells supplemented with TQ- or Met-only showed increased cell viability, while TQ coadministrated with Met had an even stronger effect on cell viability, which indicated a synergistic effect (Figure 1B).

### TQ and Met prevent ethanol-induced dysregulation of intracellular free [Ca^2+^] homeostasis

**Figure 2:**
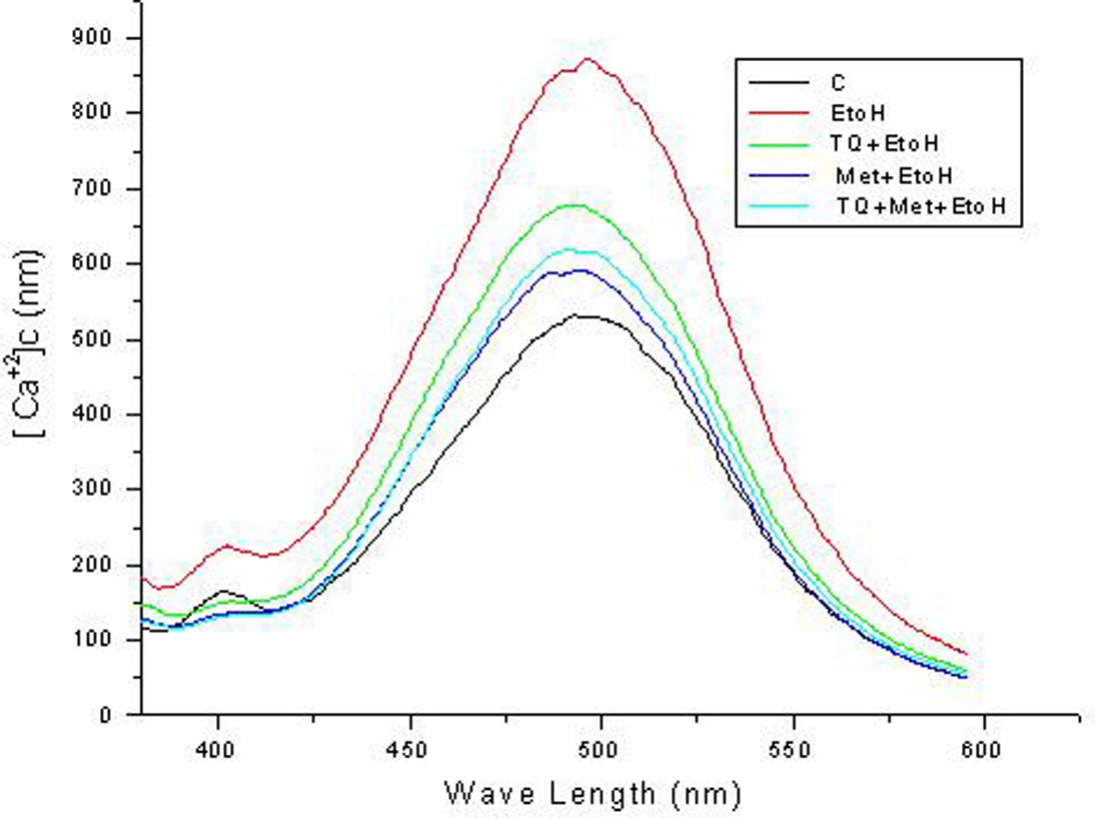
**Effect of Ethanol on elevation of cytosolic free [Ca^2+^]_c _in primary cortical neurons**. Cells were treated for 12 h with normal media as control (C), ethanol (EtOH, 100 mM), TQ (25 μM) plus ethanol (TQ+EtOH), Met (10 mM) plus ethanol (Met+EtOH), TQ plus Met plus ethanol (TQ+Met+EtOH), respectively. TQ and Met were cotreated with (100 mM) ethanol for 12 h, which was followed by Fura-2 AM labeling. The fluorescence spectra for [Ca^2+^]_c _were measured with a luminescence spectrophotometer. Different groups are indicated with respective colors by line in the representative spectra. The uppermost Red line in the spectra indicate ethanol-elevation in [Ca^2+^]_c _level compared with the other respective control. Spectra represent means ± SEM of triplicate samples (n = 3) and represent at least one of three independent experiments.

One potential mechanism of ethanol-induced neurotoxicity is through the dysregulation of cytoplasmic Ca^2+ ^concentration. Cytoplasmic Ca^2+ ^concentration governs neurotransmitter release and neural development, while its dysregulation results in neuronal cell death [4]. Abnormalities in Ca^2+ ^homeostasis, particularly overload, was linked to neural apoptosis in the present study. Cytosolic free Ca^2+ ^was measured with a fluorescent Ca^2+ ^indicator, Fura-2, after a 12 h drug treatment. Ethanol treatment increased cytosolic free Ca^2+ ^with peak levels at 869 ± 16.37 nM compared with control having peak level 531 ± 8.06 nM. TQ and Met cotreatment with ethanol restored normal Ca^2+ ^levels with a peak levels 677 ± 6.19 nM and 590 ± 5.54 nM respectively. TQ and Met in combination also prevented ethanol-induced Ca^2+ ^elevation with a peak level 617 ± 4.32 nM (Figure 2). We observed that the intracellular Ca^2+ ^concentration was significantly elevated in cells exposed to ethanol (100 mM) for 12 h compared to control, which led to neuronal cell death. TQ and Met reversed this effect that ethanol had on intracellular Ca^2+^, which suggested that they have a protective role in maintaining Ca^2+ ^homeostasis.

### TQ and Met maintain mitochondrial transmembrane potential (Δψ_M_) and prevent neuronal cell death

**Figure 3:**
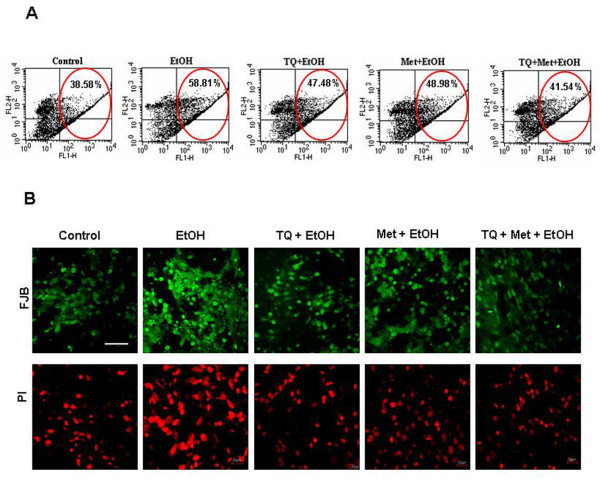
**Met and TQ prevented ethanol destabilization of mitochondrial membrane potential**. **(A) **Flow cytometric analysis of mitochondrial membrane potential (Δψ_M_) was made with JC-1. Mitochondrial polarization was monitored by flow cytometric analysis of JC-1 stained cells that were cotreated for 12 h with ethanol (EtOH, 100 mM), TQ (25 μM) plus ethanol (TQ+EtOH), Met (10 mM) plus ethanol (Met+EtOH), TQ plus Met plus ethanol (TQ+Met+EtOH) and untreated (Control). A representative collapse in Δψ_M _is associated with high FL1 fluorescence (green) and low FL2 fluorescence (red). The number in each quadrant indicates cell population in that quadrant as the percentage of total cell population. Loss of Δψ_M _was associated with an increase in FL1 fluorescence. Quantification of cells with Δψ_M _(as the percentage of total cell population) induced by ethanol, TQ and Met in different combinations as detected by flow cytometry. Data are the mean ± SEM of three independent experiments (*n = *3). The details of procedures are mentioned in materials and methods section. **(B) **Fluorescence analysis of neurodegeneration in primary cultures of fetal rat brain cortical neurons. Cultures were exposed to growth medium (Control) and with ethanol (EtOH, 100 mM), TQ (25 μM) plus ethanol (TQ+EtOH), Met (10 mM) plus ethanol (Met+EtOH), TQ plus Met plus ethanol (TQ+Met+EtOH) supplements for 12 h before staining with Fluoro-Jade B (FJB; green) and propidium iodide (PI; red). Confocal micrographs of Fluoro-jade-B and PI staining show neurodegeneration in primary cortical neurons. Magnification with 40× objective field, Scale bar = 20 μm.

We examined mitochondrial membrane potential (Δψ_M_) using flow cytometry that measured the intensity of green and red fluorescence. We used a computer-based program that calculated the ratio of green to red fluorescence after 12 h of drug treatment. Ethanol (EtOH) treatment resulted in a higher ratio of green/red FL1 fluorescence 58.8 ± 1.96% (P < 0.05) relative to control 38.5 ± 1.81%. Lower FL1 fluorescence, relative to ethanol-alone group, for TQ+EtOH 47.4 ± 1.55% (P < 0.05), Met+ EtOH 48.9 ± 2.31% (P < 0.05) or TQ+Met+EtOH 41.5 ± 1.14% (P < 0.05) indicated that Met and TQ block apoptosis by preventing Δψ_M _disruption (Figure 3A). TQ and Met resulted in the appearance of a remarkable subpopulation of cells with normal Δψ_M_, which further supported the protective effect of TQ and Met against ethanol. Fluoro-Jade-B (FJB) and propidium iodide (PI) staining was performed to assess morphological cell death induced by ethanol. After 12 h, the ethanol treatment group had a greater number of dead cells; FJB and PI staining was markedly condensed with higher intensity compared to control. Cotreatment with TQ and Met reversed the effects of ethanol (Figure 3B).

### Effect of TQ and Met on ethanol-induced changes in Bcl-2 and Bax expression in cortical neurons

**Figure 4:**
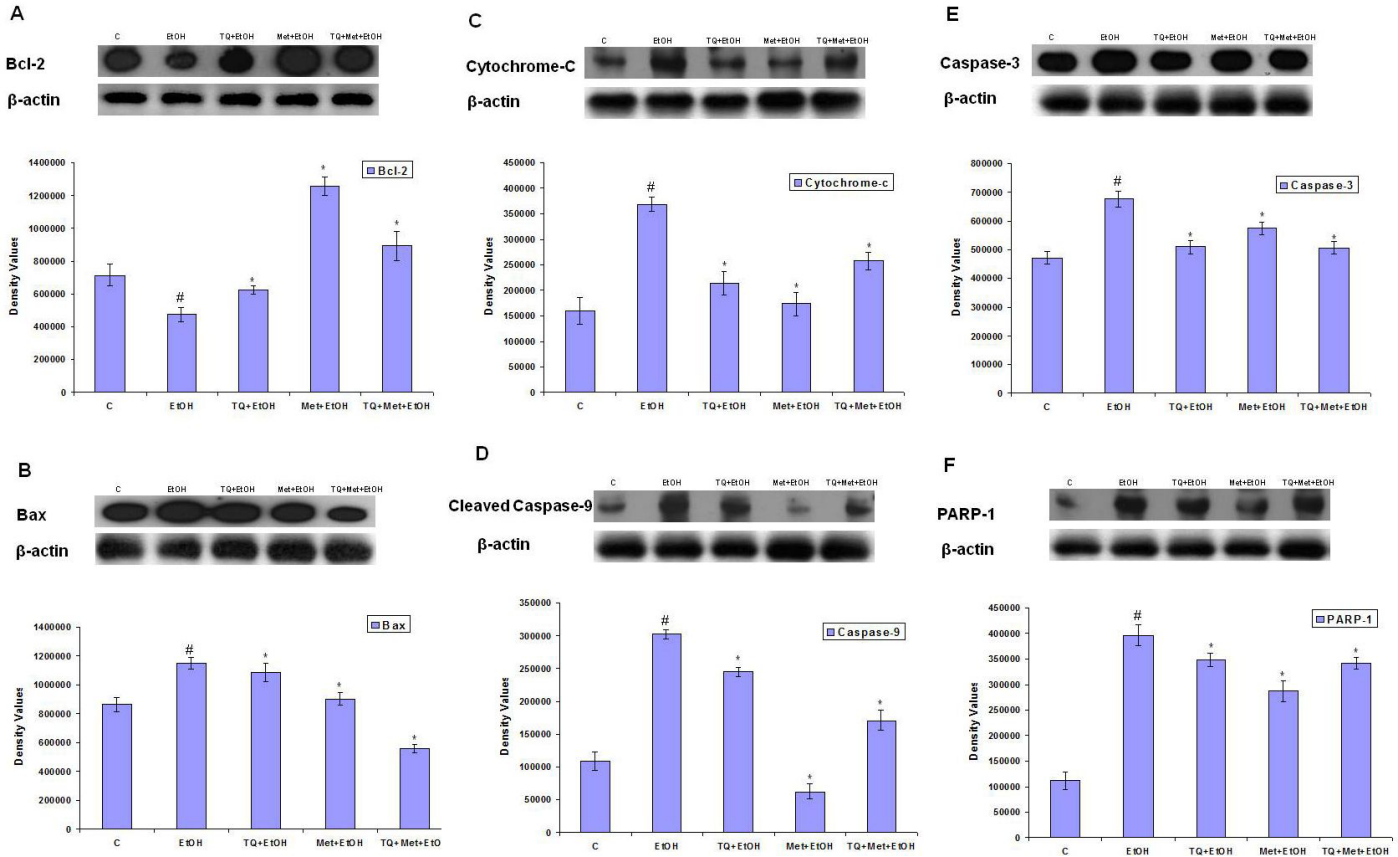
**Western blot analysis of apoptosis-related proteins in the primary cortical neurons at GD 17.5**. Cells were treated for 12 h with normal media as control (C), ethanol (EtOH, 100 mM), TQ (25 μM) plus ethanol (TQ+EtOH), Met (10 mM) plus ethanol (Met+EtOH), TQ plus Met plus ethanol (TQ+Met+EtOH), respectively. For proteins samples, we used the same drug treatment i.e., TQ and Met were cotreated with (100 mM) ethanol for 12 h. β-actin is the loading control in each case **(A) **Immunoblots of Bcl-2 and **(B) **Bax **(C) **cytochrome-c **(D) **cleaved caspase-9 **(E) **caspase-3 **(F) **cleaved PARP-1. Immunoblots are also shown with their respective histograms. Density values were expressed as mean ± SEM (n = 4) of the corresponding proteins and expressed as arbitrary units. Detail procedures are mentioned in materials and methods section. ^#^*P *< 0.05 significantly different from control; *P < 0.05 different from ethanol.

Bcl-2 and Bax are members of a family of cytoplasmic proteins that regulate apoptosis. To observe the protective effect of TQ and Met against ethanol-induced apoptosis, we assayed Bcl-2 and Bax expression levels by western blot analysis. Western blot analysis demonstrated that the expression of anti-apoptotic Bcl-2 was enhanced by TQ and Met treatment but reduced by ethanol (Figure 4A). In contrast, the pro-apoptotic Bax was upregulated by ethanol but downregulated when cotreated with TQ, Met or TQ plus Met (Figure 4B). Our results showed that TQ and Met prevented neuronal apoptosis by decreasing the expression of pro-apoptotic Bax protein while increasing the expression of anti-apoptotic Bcl-2 protein in primary cortical neurons.

### TQ and Met reduced the release of cytochrome-c into the cytosol and inhibited caspase-9 activation induced by ethanol

The mitochondrial apoptotic cascade requires the release of cytochrome-c, an intermitochondrial membrane protein, into the cytosol. This release activates caspases-9 and caspase-3, which causes neuronal cell death [27]. Our results revealed that ethanol exposure increased the expression cytosolic cytochrome-c levels relative to control. Interestingly, TQ and Met supplementation reversed this trend. Cells treated with ethanol along with TQ or Met had significantly reduced cytochrome-c expression levels relative to the ethanol treatment group (Figure 4C). Caspases are critical mediators of apoptosis in mammalian cells [28]. We analyzed the expression of cleaved caspase-9 after ethanol exposure. Our results showed that ethanol significantly increased cleaved caspase-9 in primary cortical neurons relative to the control group, while cotreatment of ethanol with TQ or Met resulted in significantly lower cleaved caspase-9 levels compared to the ethanol treatment group (Figure 4D).

### Effect of TQ and Met on ethanol-induced expression of caspase-3 and cleavage of PARP-1 in primary rat cortical neurons

Caspase-3 is one of the key executioners of apoptosis and is responsible, either partially or completely, for the proteolytic cleavage of many key proteins [28]. Furthermore, the involvement of caspase-3 in cell death was analyzed by western blotting. Ethanol induced the activation of caspase-3, while TQ and Met effectively decreased ethanol-induced expression of caspase-3 in primary cortical neurons (Figure 4E). Furthermore, we determined whether the elevated levels of activated caspase-3 that were observed in the cortical neurons treated with ethanol led to the cleavage of PARP-1, which occurs during apoptosis. Despite its function in DNA repair, overactivation of PARP-1 during neuronal excitotoxicity induces cell death [29]. The level of cleaved PARP-1 in ethanol-treated cortical neurons was significantly enhanced compared to control neurons. In the setting of ethanol, TQ and Met treatment resulted in a remarkable decrease in the level of PARP-1 and decreased its 89 kDa cleaved products in the cortical neurons (Figure 4F). Our results collectively demonstrated that an overall increase in PARP-1 cleavage occurred in the cortical neurons after exposure to ethanol. TQ and Met significantly reduced the levels of ethanol-induced cleaved PARP-1 relative to the control groups (Figure 4F).

### Effect of TQ and Met treatment on ethanol-induced neurodegeneration in the primary cultured cortical neurons after 12 h

**Figure 5:**
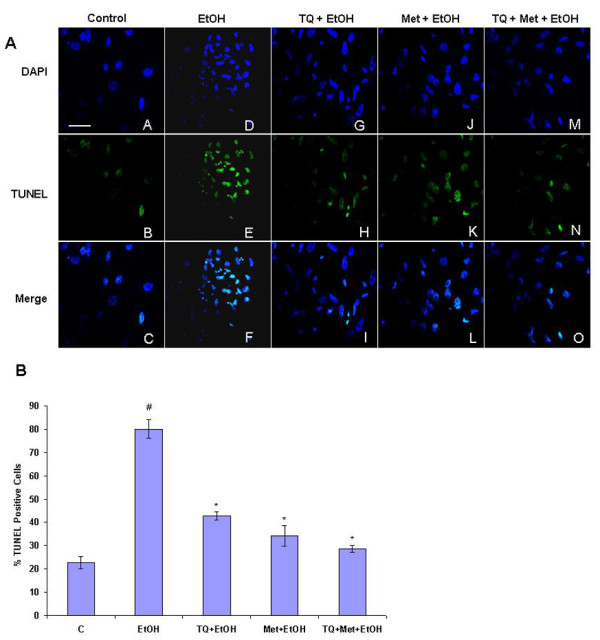
**Morphological assessment of ethanol-induced apoptosis via TUNEL assay**. **(A) **Cells were treated for 12 h with normal media as control (C), ethanol (EtOH, 100 mM), TQ (25 μM) plus ethanol (TQ+EtOH), Met (10 mM) plus ethanol (Met+EtOH), TQ plus Met plus ethanol (TQ+Met+EtOH), respectively. Effects of ethanol, TQ plus ethanol, Met plus ethanol and TQ plus Met plus ethanol on apoptotic death in prenatal rat cortical neurons was visualized with TUNEL and DAPI stains. Representative photomicrographs of TUNEL staining show apoptotic neurons after ethanol administration followed by TQ and Met. TQ and Met treatment effectively blocked ethanol-induced apoptosis, as evidenced from the lack of TUNEL-positive cells. Panels A-O display TUNEL-stained cells observed by confocal microscopy at higher magnifications with a 40× objective field, Scale bar = 20 **(B): **The percentage of TUNEL positive cells in each case were counted and the cumulative data from three independent experiments is shown here as mean ± SEM (n = 3). ^#^*P *< 0.05 significantly different from control; *P < 0.05 different from ethanol.

DNA damage is one of the main features of apoptosis. Visualization of DNA damage is accomplished with TUNEL staining, which is an assay for DNA breaks based on enzymatic labeling of free 3' DNA ends. The nuclear morphology of apoptotic neuronal cells was evaluated with TUNEL counterstained with DAPI (TUNEL: DNA fragmentation, DAPI: nuclear marker). Ethanol exposure increased the number of TUNEL-positive neurons by (58%) compared with control. TQ with ethanol showed significantly reduced the TUNEL positive cells by (38%) compared to the ethanol treatment group. Further Met with ethanol reduced the percentage of apoptotic cells by (37%) and TQ plus Met with ethanol (52%) compared to the cells treated with ethanol. Although there are DAPI-positive cells in control and ethanol-treated group, the pattern of staining differs between the groups. Ethanol-treated cells exhibited shrunken nuclei and abnormal cell morphology (Figure 5).

## Discussion

Ethanol exposure during prenatal development causes a wide range of structural and functional brain abnormalities [30,31]. It has already been established that ethanol depletes many cell populations during development, but not all populations of neurons in the brain are susceptible; furthermore, ethanol increases neuronal apoptosis, which causes malformations, including the loss of brain mass [1,32,33]. Ethanol increases ROS generation, which can trigger apoptotic cell death pathways through the activation of caspases [34-36]. The present study provides strong evidence for a direct neuroprotective role of Met and TQ. The current investigation indicated that cotreatment of ethanol with Met, TQ or Met plus TQ protects cortical neurons from the apoptosis triggered by ethanol during early development.

The evidence regarding neuroprotection with the antidiabetic drug Met is supported by studies that state its beneficial effects in various models of toxicity. It was reported that Met can protect against neurotoxicity, in addition to its antidiabetic effect, and it has a number of important, diverse functions, including in polycystic ovarian syndrome, induction of osteoblasts via AMP-activated protein kinase (AMPK) signaling pathway along with subsequent enhancement of nitric oxide synthase (eNOS) and bone morphogenetic-2 protein (BMP-2) production [37,38]. Met modulates AMPK and mitogen-activated protein kinase (MAPK) pathways to induce bone formation, which mimics the intracellular metabolic changes associated with nutritional deprivation [39,40]. An additional target of Met is the respiratory chain complex I of the mitochondria, which activates AMPK via changes in mitochondrial reactive nitrogen species [41,42]. However, we provide here a new and potential mechanism that supports a protective role of Met on apoptosis that is likely due to it maintaining mitochondria integrity and reducing [Ca^2+^]_c _overload as well as subsequent cell death [18].

Furthermore, we studied TQ, which is a natural compound with different beneficial pharmacologic effects against various cytotoxic insults. These beneficial effects have been observed *in vitro *and *in vivo *with degeneration caused by L-dopa in SH-SY5Y human neuroblastoma cells, serum/glucose deprivation induced cell death (SGD) and chronic toluene exposure [21,22,43]. In general, previously published reports showed that TQ mainly functions through its antioxidant mechanism, and it has been used as a protective agent in multiple toxicity models, such as doxorubicin-induced cardiotoxicity, cisplatin-induced nephrotoxicity and ischemic neuronal cell death [21,23,44].

We found that administration of Met and TQ in selected doses has the capability to maintain normal cell viability against ethanol. TQ, Met or a combination of both reduced the cell death induced by ethanol (Figure 1A, B). Calcium homeostasis is an important for normal cellular physiological function, and even a minute variation in normal levels can provoke abnormal cellular function and cell death [45]. This [Ca^2+^]_c _overload further disrupts mitochondrial membrane potential (Δψ_M_), which leads to neuronal apoptosis. In this study, we observed a two-fold increase in [Ca^2+^]_c _levels upon ethanol treatment compared to control cells. This cellular accumulation of calcium possibly induced cell death. Previously, a similar increase in intracellular [Ca^2+^]_c _was observed after a short (20 min) exposure to ethanol in prenatal primary neuronal cells [4], fetal hypothalamic cells and cervical ganglion neurons [45,46]. TQ and Met reduced elevated [Ca^2+^]_c _levels (Figure 2). TQ and Met modulate and stabilize Δψ_M_. Our findings that showed ethanol-induced alterations in mitochondrial function were also in agreement with a recent study that demonstrated that vitamin E and BDNF have neuroprotective and modulating roles during ethanol toxicity in cerebellar granule cells. The mechanism of neuroprotection was similar to Met and TQ involved the stabilization of mitochondrial membrane potential and inhibition of apoptotic cascade [12].

The Bcl-2 protein family plays an important role in apoptotic signal transduction by regulating mitochondrial function [47]. It was reported that mitochondrial cytochrome-c is released into the cytosol through the permeability transition pore, which is regulated by Bcl-2 family proteins [47]. The pro-apoptotic protein Bax destabilizes Δψ_M _and facilitates cytochrome-c release [48], while the anti-apoptotic protein Bcl-2 functions to restore membrane potential and block cytochrome-c release. In our findings, we found that TQ and Met increased Bcl-2 levels in primary cortical neurons exposed to ethanol. TUNEL and western blot analysis demonstrated that TQ and Met cotreatment protects neurons by lowering the expression of caspase-3, cytochrome-c, cleaved caspase-9 while also increasing the expression of Bcl-2 compared to cells treated with ethanol alone. This finding implied that TQ and Met potentially prevents apoptosis by regulating the mitochondrial path way.

PARP-1 functions in apoptosis by acting as a survival factor and a death promoter, depending on the duration and severity of DNA damage [49]. We observed cleavage of PARP-1 to its respective 89 kDa fragment after a 12 h exposure to ethanol. In Met and TQ treatment groups, fragmentation of PARP-1 was significantly reduced. In support of our results, other investigators noted caspase-dependent cleavage of PARP-1 that was induced by an ethanol concentration (4.0 mg/ml), similar to the one used in this study, for 0, 4, 12 and 24 h time periods [50]. The potential benefit of Met and TQ on primary neuronal cell viability was evaluated by TUNEL assay. Met and TQ decreased DNA damage and reduced the number of TUNEL-positive cells produced by ethanol treatment. A study using an analogous experimental approach demonstrated similar DNA damage induced by ethanol in cultured cortical neurons [50].

The present findings supported a conclusion that ethanol disrupts Δψ_M_, sustains Ca^2+ ^overload and triggers an apoptotic cascade. Met and TQ inhibition of ethanol-induced cell death in primary rat cortical neurons might occur via an antioxidant mechanism that maintains mitochondrial integrity. Since Met and TQ are safe and nontoxic, more studies should be conducted to explain their synergistic actions with other drugs.

## Conclusion

In summary, this study demonstrated that TQ and Met exert a neuroprotective effect by decreasing ethanol-mediated mitochondria-dependent apoptosis. Although additional studies are needed to determine the molecular mechanisms underlying TQ and Met actions, TQ and Met are readily available and safe agents, so they could eventually be used as neuroprotectants against ethanol-induced neurodegeneration during early development.

## Methods

### Animal treatment

Female (*n *= 12) Sprague-Dawley rats (250 g, Gyeongsang National University, Neurobiology Laboratory, Chinju, South Korea) were housed in a temperature-controlled environment (light cycle: 06:00-20:00 h) with food ad libitum. Time of pregnancy was assigned from the day of insemination equal to gestational day (GD) 0.5. After GD 17.5 pregnant Sprague-Dawley rats were killed by decapitation and after an i.v. injection of pentobarbital sodium (3 mg/100 g body weight). Animals were treated in accordance with standard guidelines for laboratory animal care. Experimental procedures were approved by the local animal ethics committee of the Division of Applied Life Sciences, Department of Biology, Gyeongsang National University, South Korea.

### Primary cell culture and drug treatment

Cultures were prepared from the cortices of prenatal rats at GD 17.5. Pooled cortical brain tissue was treated with 0.25% trypsin-EDTA for 20 min and dissociated by mechanical trituration in ice-cold calcium-free and magnesium-free Hank's balanced salt solution (pH 7.4). After pelleting by centrifugation, cells were plated (1 × 10^6 ^cells/ml) in chamber slides or cell culture plates that were pre-coated with polylysine (0.02 g/l). Culture medium consisted of Dulbecco's modified Eagle medium (DMEM) with 10% heat-inactivated fetal bovine serum, 1 mM pyruvate, 4.2 mM sodium bicarbonate, 20 mM HEPES, 0.3 g/L bovine serum albumin, 50 U/ml penicillin, and 50 mg/L streptomycin. Cultures were maintained at 37°C in a humidified atmosphere of 5% CO_2 _and 95% air. Neuroglia cells were inhibited with media containing 100 μM Cytosine β-D-Arabino Furanoside (Sigma) for 12 h. After 4 days, cortical neurons were assigned to of five groups: 1) Control (C): cells incubated with normal DMEM medium; 2) Ethanol treatment alone (EtOH): treated with media containing 100 mM ethanol; 3) TQ plus EtOH cotreatment (concurrently); 4) Met plus EtOH cotreatment (concurrently); 5) TQ plus Met plus EtOH cotreatement (concurrently). The TQ dose (25 μM) was selected on the basis of MTT assay, and the Met dose (10 mM) was from unpublished lab data; these were used in different groups and combinations. All drug treatment groups were incubated for 12 h *in vitro *selected on the basis of preliminary experiments carried out and on the basis of that we selected 12 h as an optimum condition to carry out the final experiments reported here. In all experiments 100 mM ethanol concentration was used selected from of our previously published studies [4].

### Cell viability

The logarithmic growth phase of primary neuronal cells was taken for growth assays using 3-[4,5-dimethylthiazol-2-yl]-2,5-diphenyl tetrazolium bromide (MTT). Primary cortical neurons were seeded onto 96-well plates (1 × 10^5 ^cells/well) in 200 μl of DMEM media (control). DMEM media with ethanol (100 mM), TQ (10, 15, 25 and 35 μM) and Met (10 mM) in different combinations were incubated at 37°C for 12 h in a humidified 5% CO2 incubator. TQ and Met were cotreated with ethanol in all experimental groups. After incubation, cell viability was determined by adding MTT (5 mg/ml in phosphate buffer saline, PBS) to each well and incubating these cells for 4 h at 37°C. Formazan dissolved in organic solvent was added, to the cells. Plates were placed on a shaker and agitated for 10 to 20 min. Plates were read at 550 to 570 nm (L1) and 620 to 650 nm (L2) on scanning microplate reader spectrophotometer; 620 to 650 nm absorbance results measured cell debris and well imperfections. The final optical density (OD) obtained (OD = L1-L2) was used to calculate the percentage of cell survival, which was expressed as absorbance treated wells/absorbance control wells × 100%.

### Intracellular free Ca^2+ ^measurement

The intracellular Ca^2+ ^concentration was measured with the fluorescent Ca^2+ ^indicator Fura-2 acetoxymethyl ester [51]. After four days of growth, cells were treated with normal media (control), ethanol (100 mM), TQ (25 μM) plus ethanol, Met (10 mM) plus ethanol, or TQ plus Met in combination with ethanol for 12 h. After treatment, cultured cortical neurons (triplicate plates; each containing 1 × 10^6 ^cells) were washed twice with Krebs buffer and then incubated in DMEM media containing 5 μM Fura-2 AM at 37°C in a humidified incubator with 5% CO_2 _for 60 min. Cells were washed twice with Locke's solution (pH 7.8), and Fura-2 fluorescence signals of [Ca^+2^]_c _were measured with a luminescence spectrophotometer (LS50B, Perkin Elmer, Boston, MA). Excitation light from a Xenon lamp was alternated between 340 and 380 nm band-pass filters and the fluorescence emitted at 510 nm was revealed by a photon-counting photomultiplier. The 340 nm/380 nm fluorescence ratio, averaged over a period of 2 s, was measured. Fluorescence signals were acquired, stored and analyzed using a computer with universal imaging software or a MicroVax II computer with origin 7 software. Intracellular calcium was determined using the ratio method as mentioned above and from the Grynkiewicz equation [52].

$$\left[\mathrm{Ca}\right]\;=\;K_{d}\;\times \;\frac{\left(R\;-\;\mathrm{Rmin}\right)}{\left(\mathrm{Rmax}-R\right)}\;\times \;\frac{\mathrm{Sf}2}{\mathrm{Sb}2}$$

Kd: dissociation constant of the Fura-2 Ca^+2 ^interaction to be 225 nM in the cytosolic environment; R: fluorescence ratio at 340 and 380 nm; Rmin: ratio with zero Ca^+2^; Rmax: ratio with saturating Ca^+2 ^(using calcium chloride); Sf2: fluorescence at 380 nm with zero Ca^+2^; Sb2: fluorescence at 380 nm with saturating Ca^+2^.

### Measurement of mitochondrial membrane potential with JC-1

Mitochondrial membrane potential (Δψ_M_) was monitored using JC-1 Δψ_M _detection kit (Biotium Inc., Hayward, CA, USA), according to the manufacturer's protocol. JC-1 emits either green or red fluorescence, depending on the Δψ_M_. A green signal indicated mitochondria depolarization, while a red signal indicated polarized mitochondria [53]. Thus, the shift from red to green fluorescence was considered a reliable indicator of a drop in Δψ_M_. After 4 days, neuronal cells were treated with normal media (control; C), ethanol (100 mM; EtOH), ethanol plus TQ (25 μM), Met plus ethanol and TQ plus Met plus ethanol in different groups and combinations. All drug treatment groups were incubated for 12 h *in vitro *at the same time at 37°C. After drug treatment, cells in triplicate culture were washed with PBS twice, harvested and stained with JC-1 reagents at 37°C for 15 min. Cells were resuspended twice in 1× assay buffer. JC-1 aggregates in healthy mitochondria emit red fluorescence at 590 nm. JC-1 monomers that leaked from stressed mitochondria emit green fluorescence at 530 nm. Red and green fluorescence were measured in the green (FL-1) and red (FL-2) channels of a flow cytometer. Changes in Δψ_M _were measured at the single-cell level with a FACS Caliber flow cytometer (Becton Dickinson, San Jose, CA, USA). In total, 10,000 cells were acquired for analysis by Cell Quest software, version 3.0 (Becton Dickinson, San Jose, CA, USA), and quantification of cells with low Δψ_M_, as the percentage of total cell population, was performed.

### Western blotting

Western blot analysis was done as previously described with some modifications [54]. Immunoreactions were performed with primary antibodies: Bax and Bcl-2 (Rabbit polyclonal, Santa Cruz Biotechnology; 1:1000, 24 h, 4°C), cytochrome-c (Goat, Santa Cruz Biotechnology; 1:1000, 24 h, 4°C), PARP-1 (Mouse monoclonal, Santa Cruz Biotechnology; 1:1000, 24 h, 4°C), caspase-3 and caspase-9 antibody (Rabbit, Cell signaling 1:1000 24 h, 4°C). After rinsing the blots, horseradish peroxidase-conjugated goat anti-mouse, mouse anti-goat or goat anti-rabbit IgG-HRPs (Santa Cruz Biotech 1:1000) were incubated with the blots for 2 h at room temperature. Immunoreactions were also performed using β-actin antibody (Cell Signaling) as loading controls. Proteins were detected by chemiluminescence using an ECL-detecting reagent (Amersham Pharmacia Biotech, western blotting detection reagents) with the manufacturer's protocol. Chemiluminescent blots were then exposed to X-ray film. Exposed X-ray film was scanned, and optical densities were analyzed by densitometry using the computer-based SigmaGel, version 1.0 (Jandel Scientific, San Rafeal, Chicago, USA).

### TUNEL and DAPI staining

To assay apoptosis, cells were stained with TUNEL (GenScript Corporation, USA) and counterstained using 4',6-diamidino-2-phenylindole (DAPI). In situ detection of apoptotic cell death was performed using terminal deoxynucleotidyl transferase (TdT)-mediated dUTP nick end-labeling (TUNEL) in cortical neuron cultures as previously done with a little modification [13]. TUNEL staining was performed, according to the supplier's recommendations, using the In Situ Cell Death Detection kit for Fluorescein (GeneScript, USA). Cells were also incubated with DAPI (Molecular Probes, Eugene, OR, USA) for 10 min at room temperature and then rinsed with distilled water. Glass cover slips were mounted on glass slides with mounting medium. A DAPI filter was used to detect DAPI staining (blue color), and an FITC filter was used was to detect TUNEL staining (green color). TUNEL-positive (green) and DAPI-positive (blue) staining patterns were acquired with a confocal laser scanning microscope (Fluoview FV 1000, Olympus, Japan). TUNEL-positive cells in the different regions of each slide were counted by an observer who was blinded to the treatment conditions.

### Fluoro-Jade-B (FJB) and PI staining

Fluoro-Jade-B (FJB) staining was performed as previously described [55]. Immunofluorescence was performed on GD 17.5 primary cortical neuron cultures grown *in vitro *on poly-D-lysine-coated chamber slides. Four-day-old cultures were treated for 12 h at 37°C as indicated in the figure legends. Treated cultures were fixed for 5 min with 4% paraformaldehyde in PBS and stored at -70°C. Slides were air dried for 3 h and then subjected to the following treatments in order: 10 min in 0.06% potassium permanganate solution, distilled water rinse, 20 min in 0.1% acetic acid containing 0.0004% FJB (Calbiochem, San Diego, California, USA) and three washes in distilled water. The slides were allowed to dry at 55°C for 10 min and viewed under an FITC filter in a confocal microscope (Olympus Fluoview FV1000, Japan). For propidium iodide (PI) staining, slides were dipped in PI solution (1 μg/ml) in PBS with gentle mixing for 20 min at room temperature and washed twice with PBS for 10 min. Glass cover slips were mounted on the slides with mounting medium.

### Data analysis and statistics

The objective bands on western blots were scanned and analyzed by densitometry using a computer with the SigmaGel System (SPSS Inc., Chicago, IL). Density values were expressed as mean ± SEM. One-way ANOVA followed by Tukey-Kramer multiple comparisons test was performed to determine the significance of differences between relevant treatment groups. In every case, the acceptance level for statistical significance was *P < 0.05*.

## Abbreviations

Met: Metformin; TQ: Thymoqinone; FAS: fetal alcohol syndrome; PTZ: pentylenetetrazol; GD: gestational days; DEPC: diethyl pyrocarbonate; NBP: 4% neutral buffer paraformaldehyde; DMEM: Dulbecco's modified Eagle medium; MTT: 3-[4,5-dimethylthiazol-2-yl]-2,5-diphenly tetrazolium bromide; PCD: programmed cell death; PTP: permeability transition pore; NS: *Nigella sativa*.

## Authors' contributions

IKU carried out the experiments and drafted the manuscript. NAU, MIN and HYL helped in analysis and drafting the manuscript. MOK wrote discussion and conceived of the study and interpreted the data and discussion of the results. All the authors approve the final manuscript.
